# The association between maternal diabetes and the risk of epilepsy in offspring: a systematic review and meta-analysis

**DOI:** 10.3389/fendo.2026.1960502

**Published:** 2026-09-11

**Authors:** Huan Zeng, Daying Long, Yefang Geng, Xun Li, Hua Zhou, Xiaojing Liu, Yunhong Lei

**Affiliations:** 1 Department of Interventional Radiology, Affiliated Central People’s Hospital, China Three Gorges University, Yichang, Hubei, China; 2 Department of Interventional Radiology, Yichang Central People’s Hospital, Yichang, Hubei, China; 3 Department of Interventional Radiology, China Three Gorges University, Yichang, Hubei, China; 4 Department of Hepatobiliary, Pancreatic and Splenic Surgery, The Central Hospital of Enshi Tujia and Miao Autonomous Prefecture (Enshi Clinical College of Wuhan University), Enshi, Hubei, China; 5 Department of Interventional Diagnosis and Therapy, Yichang Yiling Hospital, Yichang, Hubei, China; 6 Department of Radiology, Zhijiang People’s Hospital, Zhijiang, Hubei, China; 7 Department of Gastroenterology, Yidu People’s Hospital, Yidu, Hubei, China; 8 Tannks, Yantai Yuhuangding Hospital, Yantai, Shandong, China; 9 Tannks, Yichang Hubo Medical Research Institute, Yichang, Hubei, China; 10 Tannks, Wuhan Zhongjian Life Science Research Institute, Wuhan, Hubei, China

**Keywords:** epilepsy, gestational diabetes mellitus, maternal diabetes, meta-analysis, type 1 diabetes mellitus, type 2 diabetes mellitus

## Abstract

**Background:**

Epilepsy is among the most prevalent neurological disorders in children, with nearly half of all cases remaining idiopathic. The global prevalence of maternal diabetes—encompassing both pregestational types (type 1 diabetes mellitus (T1DM) and type 2 diabetes mellitus (T2DM)) and gestational diabetes mellitus (GDM)—has been steadily increasing, raising growing concerns regarding its impact on offspring neurodevelopment. While accumulating evidence suggests that maternal diabetes is associated with an elevated risk of various neurodevelopmental disorders in children, its specific relationship with the risk of epilepsy remains unclear, with available data being both limited and conflicting. To address this gap, we conducted a meta-analysis to comprehensively evaluate the association between distinct types of maternal diabetes (pregestational and gestational) and the subsequent risk of epilepsy in offspring.

**Methods:**

We systematically searched the PubMed, Embase, Web of Science, and the Cochrane Library for observational studies examining the association between maternal diabetes and offspring epilepsy risk, covering the period from database inception to July 2026. Two independent reviewers performed literature screening, data extraction, and risk-of-bias assessment. Statistical analyses were performed using Stata version 14.0.

**Results:**

A total of three studies, encompassing 5,287,837 participants, met the inclusion criteria. Pregestational diabetes—both T1DM and T2DM—was significantly associated with an increased risk of epilepsy in offspring (T1DM: HR = 1.33, 95% CI [1.17, 1.52], *P* < 0.001; T2DM: HR = 1.38, 95% CI [1.25, 1.51], *P* < 0.001). Nevertheless, owing to the observational design of the included studies, these associations should not be interpreted as causal. Regarding GDM, the pooled estimate did not reach conventional statistical significance (HR = 1.04, 95% CI [0.93, 1.16], *P* = 0.469). Furthermore, sensitivity analyses revealed that this null finding lacked robustness, with the pooled estimate being substantially influenced by individual studies. Consequently, the current evidence is insufficient to either support or refute a conclusive association between GDM and offspring epilepsy, and any positive findings must be interpreted with considerable caution.

**Conclusions:**

This meta-analysis suggests that pregestational T1DM and T2DM may increase the risk of epilepsy in offspring. In contrast, for GDM, the available analytical evidence remains inconclusive and displays notable between-study heterogeneity; although the summary effect was not statistically significant, it proved highly unstable under sensitivity testing. Given the limited number of included studies and the inability to fully exclude residual confounding, the above findings—particularly those pertaining to GDM—should be regarded as exploratory rather than definitive causal inferences. Interpretation of these results warrants prudence. Future large-scale, high-quality prospective cohort studies are urgently needed to validate these observations, with special emphasis on stringent glycemic management in pregnant women with diabetes and long-term neurodevelopmental surveillance of their offspring.

**Systematic review registration:**

https://www.crd.york.ac.uk/PROSPERO/view/CRD420261465624, identifier CRD420261465624.

## Introduction

Epilepsy remains one of the most prevalent neurological disorders in children (1, 2). From 1990 to 2021, the absolute number of prevalent cases among children and adolescents increased by 27.75%, alongside a modest rise in prevalence rates (3). The etiology of epilepsy is complex and multifaceted; however, in approximately half of all cases, the underlying causes remain unknown to date (4). In recent years, the potential influence of maternal hyperglycemia during pregnancy on fetal brain development has garnered increasing attention and has emerged as a key research focus in perinatal epidemiology.

Maternal diabetes represents one of the most common metabolic complications during pregnancy and is broadly categorized into three distinct subtypes: pregestational type 1 diabetes mellitus (T1DM), pregestational type 2 diabetes mellitus (T2DM), and gestational diabetes mellitus (GDM) (5). Driven by the global obesity pandemic, delayed childbearing, and updated diagnostic criteria for diabetes, the prevalence of maternal diabetes—particularly GDM—has been rising steadily (6). It is estimated that approximately 7% to 10% of pregnancies worldwide are complicated by diabetes, with GDM accounting for over 90% of these cases (7). A national cohort study reported prepregnancy exposure rates of approximately 0.6% for T1DM, 0.2% for T2DM, and 1.6% for GDM, respectively (8). In the United States, the overall prevalence of GDM has surpassed 7% (9). Beyond increasing the risk of perinatal complications such as preeclampsia, preterm birth, macrosomia, and neonatal hypoglycemia (10), maternal diabetes has raised widespread concern regarding its potential long-term health consequences for future generations.

The fetal period constitutes a critical window for the development of brain structure and function, during which the central nervous system is exceptionally vulnerable to adverse external exposures. Through placental transfer, maternal blood glucose levels directly shape the fetal energy metabolism and endocrine milieu. Exposure to sustained hyperglycemia may disrupt normal neurodevelopmental trajectories via multiple interconnected mechanisms, including fetal hyperinsulinemia, chronic hypoxia, oxidative stress, and inflammatory responses (11, 12). Substantial epidemiological evidence has linked maternal diabetes to neurodevelopmental abnormalities in offspring, such as cognitive impairment, attention-deficit/hyperactivity disorder, and autism spectrum disorder (13). Nevertheless, the specific association between maternal diabetes and offspring epilepsy risk remains largely unclear. The pathophysiology of epilepsy involves aberrant neuronal discharges and neural network dysfunction (14), potentially arising from structural brain anomalies, metabolic disturbances, or immune-mediated inflammation (15–17). Theoretically, pregnancy-related hyperglycemia could influence epilepsy susceptibility through these mechanistic pathways.

Several studies have demonstrated that pregestational diabetes (T1DM and T2DM) significantly elevates the risk of epilepsy in offspring (8, 18, 19). Conversely, other investigations have suggested that GDM is not associated with offspring epilepsy risk (8, 18, 20). Notably, most existing studies have treated maternal diabetes as a single, undifferentiated exposure, and systematic evidence concurrently evaluating the independent associations of the three specific subtypes—T1DM, T2DM, and GDM—with offspring epilepsy remains scarce. Given the limited number of epidemiological studies and the inconsistency of their findings, a systematic synthesis and quantitative appraisal of the available evidence are urgently needed. To address this gap, the present meta-analysis was designed to separately evaluate the associations of pregestational diabetes (T1DM and T2DM) and GDM with the risk of epilepsy in offspring, with the goal of informing evidence-based clinical monitoring and public health strategies.

## Methods

This meta-analysis was conducted in accordance with the updated Preferred Reporting Items for Systematic Reviews and Meta-Analyses (PRISMA) guidelines and was registered on the PROSPERO international prospective register of systematic reviews (registration number: CRD420261465624).

### Data sources and search strategy

We systematically searched the PubMed, Embase, Web of Science and Cochrane Library databases for observational studies examining the association between maternal diabetes and offspring epilepsy risk, covering the period from database inception to July 2026. The search strategy employed a combination of Medical Subject Headings (MeSH) terms and free-text keywords, including: pregestational diabetes, gestational diabetes mellitus, type 1 diabetes mellitus, type 2 diabetes mellitus, epilepsy, and related synonyms. Optimized search expressions were tailored to the specific syntax of each database. The complete search strategy is available in the **Supplementary Material** (**Supplementary Table 1**).

### Inclusion and exclusion criteria

Studies were considered eligible if they met all of the following criteria:

1. Population: Offspring populations, with no restrictions on age, sex, or ethnicity;
2. Exposure: Maternal diabetes during pregnancy, specifically including T1DM, T2DM, or GDM, with clear diagnostic criteria;
3. Comparator: Offspring of mothers without diabetes or without documented hyperglycemia during pregnancy;
4. Outcome: Diagnosis of epilepsy in offspring, defined according to the International Classification of Diseases (ICD) coding or confirmed by qualified clinicians;
5. Study design: Observational studies (cohort or case–control designs) reporting extractable effect estimates, including relative risks (RR), hazard ratios (HR), or odds ratios (OR), along with their 95% confidence intervals (CIs), or providing sufficient raw data to compute these measures.

Studies were excluded if they met any of the following criteria:

1. Failure to clearly distinguish between distinct subtypes of diabetes (i.e., combining pregestational diabetes with GDM or not specifying subtypes);
2. Duplicate publications or overlapping data (in such cases, only the study with the larger sample size or longer follow-up period was retained);
3. Reviews, commentaries, conference abstracts, case reports, animal studies, or articles for which the full text was unavailable.

### Data extraction

Literature screening and data extraction were independently performed by two researchers. Disagreements were resolved through discussion; if consensus could not be reached, a third researcher was consulted, or the original authors were contacted for additional information. For each included study, the following data were independently extracted: (1) basic citation information: first author, publication year, country; (2) study design and data source; (3) study period and duration of follow-up; (4) total sample size and distribution of participants across exposure and non-exposure groups; (5) type of maternal diabetes exposure (T1DM, T2DM, or GDM); (6) list of covariates adjusted for in the analyses; and (7) effect sizes (HR, RR, or OR) with corresponding 95% CIs for each exposure type.

### Risk of bias

Two researchers independently evaluated the methodological quality of the included observational studies using the Newcastle-Ottawa Scale (NOS). This scale consists of three domains: Selection (4 items), Comparability (1 item, with a maximum of 2 points), and Outcome (for cohort studies) or Exposure (for case–control studies) (3 items), yielding a total possible score of 9 points. Studies scoring 7–9 were classified as high quality, 5–6 as moderate quality, and ≤4 as low quality. Any scoring disagreements were resolved through consensus discussion.

### Data synthesis and analysis

All statistical analyses were performed using Stata version 14.0. Pooled effect estimates were calculated using HRs and their 95% CIs as the primary summary measures. Between-study heterogeneity was evaluated using Cochran’s Q test and the I² statistic. A fixed-effect model was applied when I² < 50% and *P* > 0.10 (21); conversely, a random-effects model was used when I² ≥ 50% and *P* ≤ 0.10 (22). Separate meta-analyses were conducted for each of the three exposure types (T1DM, T2DM, and GDM). Due to the limited number of included studies and insufficient data for stratified analyses, subgroup analyses were not performed; instead, potential sources of heterogeneity are elaborated in the Discussion section. Sensitivity analyses were carried out using the leave-one-out approach, in which the pooled effect size was recalculated after sequentially excluding each individual study, to determine whether the overall results were driven by a single study and to test the robustness of the findings. Publication bias was qualitatively assessed using funnel plots and quantitatively evaluated using Egger’s test and Begg’s test.

## Results

### Literature search and selection

**Figure 1:**
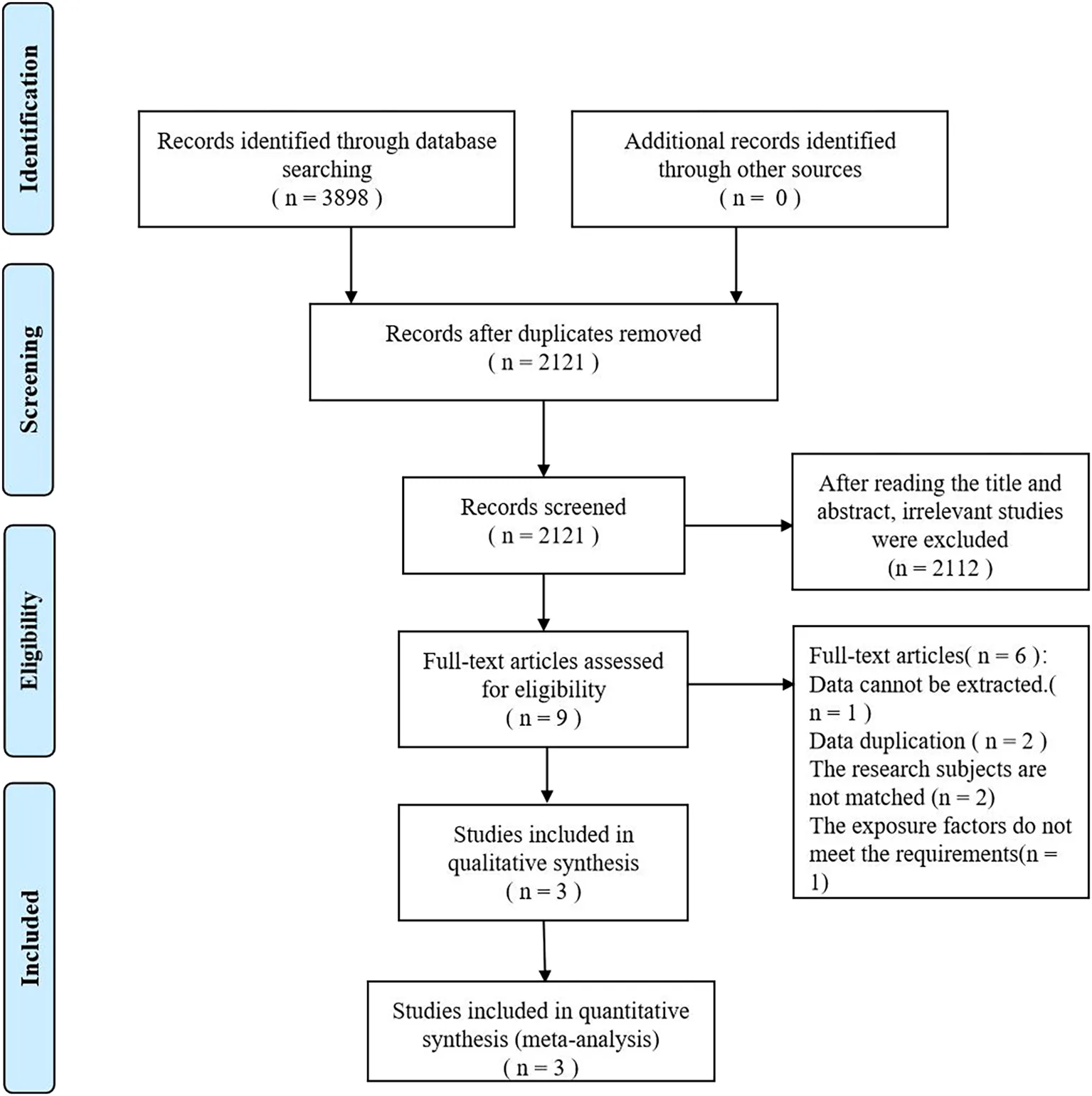
Flowchart of study selection.

The initial database search yielded a total of 3,898 records. After removing duplicates using EndNote software, 2,121 records remained. Two researchers independently screened the remaining records against the prespecified inclusion and exclusion criteria. First, 2,112 irrelevant records were excluded based on title and abstract review. Subsequently, the full texts of the remaining 9 articles were retrieved and assessed in detail for eligibility. Six articles were excluded at this stage, ultimately leaving three studies (8, 18, 19) that met all inclusion criteria (**Figure 1**).

### Characteristics of eligible studies

The baseline characteristics of the included studies are summarized in **Table 1**. This meta-analysis incorporated three observational studies, all of which were retrospective cohort designs, published between 2023 and 2026. These studies were conducted across three countries: China, Canada, and Sweden. The total sample size encompassed 5,287,837 participants. Regarding the study periods, the birth cohorts ranged from 2002 to 2021, with follow-up extending through 2015 to 2023. In terms of exposure classification, all three studies simultaneously evaluated the three distinct subtypes—pregestational T1DM, pregestational T2DM, and GDM—as separate exposures. With respect to covariate adjustment, each study controlled for a different set of confounders; all studies adjusted for maternal age and offspring sex, while the majority additionally adjusted for perinatal factors such as maternal body mass index, parity, hypertensive disorders, and preterm birth. The methodological quality of the included studies, as assessed by the Newcastle-Ottawa Scale (NOS), ranged from 8 to 9 points, indicating high quality (**Table 2**).

**Table 1:** Baseline characteristics of included studies.

Study	Country	Study design	Data source	Period of Study	Sample size	Follow-up duration	Type of exposure	Adjusted confounding factors
Chen et al., 2023 (18)	China	Retrospective cohort study	Taiwan Maternal and Child Health Database	Born between 2004 and 2008, followed up until 2015	877233	7–12 years	T1DM,T2DM,GDM	Parental age, year of birth, child sex, family income, urbanization level, hypertensive disorders, preterm delivery
Driollet et al., 2026 (19)	Canada	Retrospective cohort study	Institute for Clinical Evaluative Sciences	Born between 2002 and 2018, followed up until 2020	2105553	10.2 years(Median)	T1DM,T2DM,GDM	Maternal age, parity, ON-Marg material deprivation index, ON-Marg ethnic diversity index, neighbourhood income index, rurality, Ontario Drug Benefit program enrollment, maternal immigration status, maternal history of neurodevelopmental disorders, child sex, parity, year of birth, pre-pregnancy obesity, pre-pregnancy hypertension
Hossin et al., 2026 (8)	Sweden	Prospective cohort study	Swedish Medical Birth Register	Born in 1998 and 2021, followed up until 2023	2305051	13 years(Median)	T1DM,T2DM,GDM	Child sex, birth year; maternal: age, parity, educational level, country of birth, cohabitation status, smoking, body mass index, alcohol use disorder, chronic hypertension, history of epilepsy, psychiatric disorders, use of antidiabetic medications; paternal: age, history of epilepsy, psychiatric disorders, diabetes (mutually adjusted for each other)

T1DM, Type 1 Diabetes Mellitus; T2DM, Type 2 Diabetes Mellitus; GDM, Gestational Diabetes Mellitus.

**Table 2:** Risk of bias assessment.

Author (year)	Selection	Comparability	Outcome/exposure	Total score	Quality
Chen et al., 2023 (18)	★★★★	★★	★★★	9	High
Driollet et al., 2026 (19)	**★★★★**	★★	★★	8	High
Hossin et al., 2026 (8)	★★★★	★★	★★	8	High

One star represents 1 point.

### Meta-analysis results

#### Association between gestational diabetes mellitus and offspring epilepsy risk

**Figure 2:**
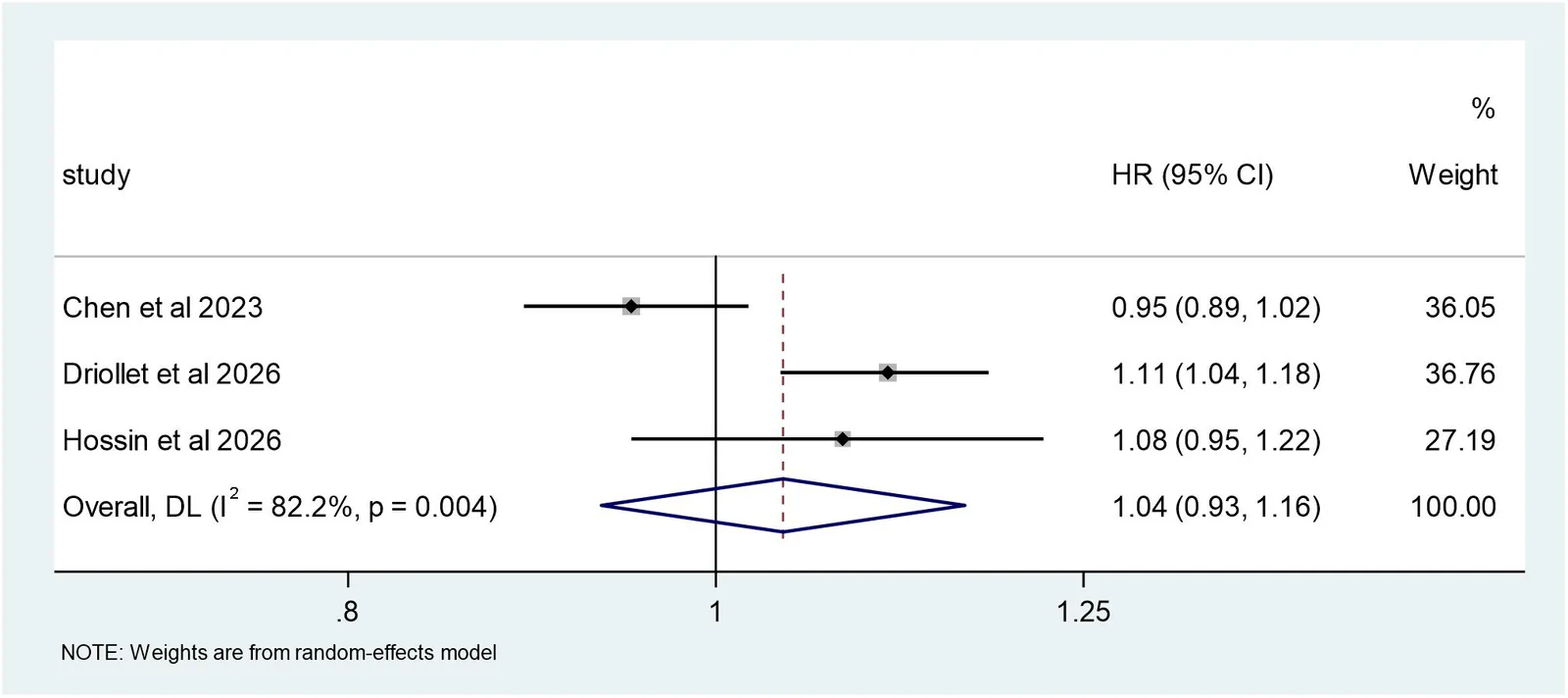
Forest plot of the association between gestational diabetes mellitus and the risk of epilepsy in offspring.

Three studies reported on the association between GDM and the risk of epilepsy in offspring. Heterogeneity testing revealed substantial between-study variation (I² = 82.20%, *P* = 0.004), prompting the use of a random-effects model. The pooled analysis demonstrated no statistically significant association between GDM and offspring epilepsy risk (HR = 1.04, 95% CI [0.93, 1.16], *P* = 0.469) (**Figure 2**).

#### Association between pregestational type 2 diabetes mellitus and offspring epilepsy risk

**Figure 3:**
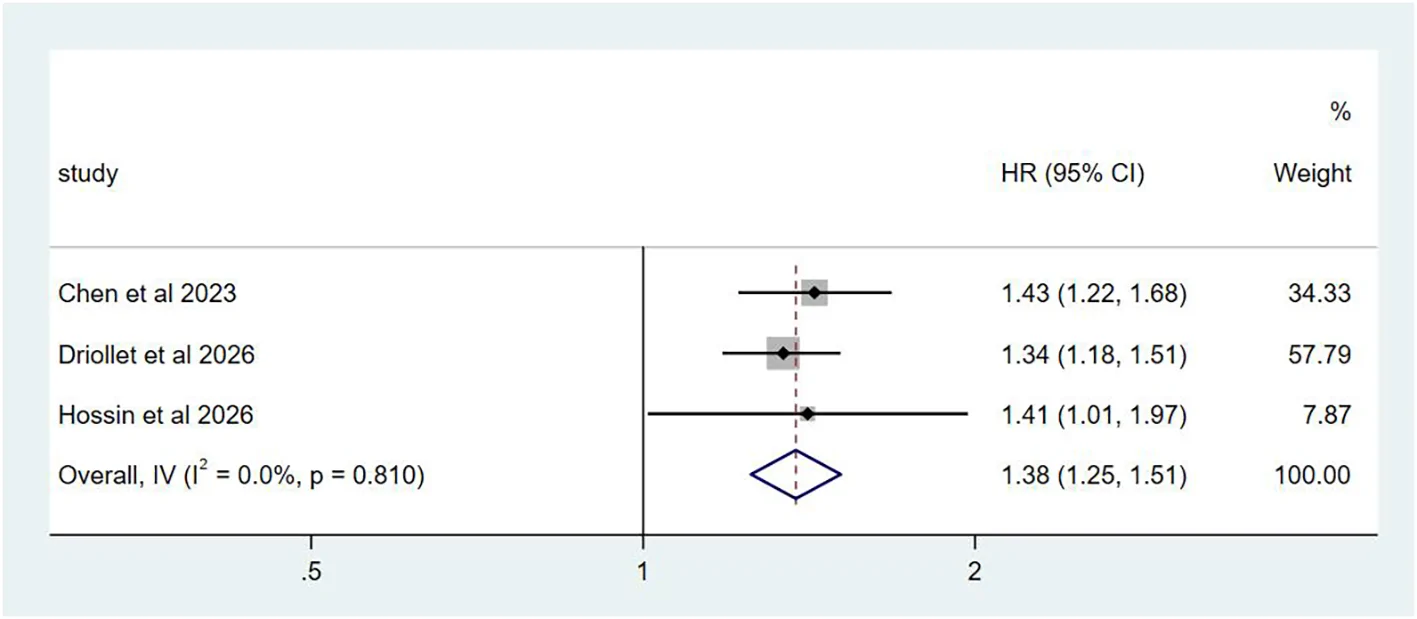
Forest plot of the association between pregestational type 2 diabetes mellitus and the risk of epilepsy in offspring.

Three studies examined the relationship between maternal T2DM and offspring epilepsy risk. Heterogeneity was negligible across studies (I² = 0%, *P* = 0.810), supporting the use of a fixed-effect model. The meta-analysis showed that pregestational T2DM significantly increased the risk of epilepsy in offspring (HR = 1.38, 95% CI [1.25, 1.51], *P* < 0.0001) (**Figure 3**).

#### Association between pregestational type 1 diabetes mellitus and offspring epilepsy risk

**Figure 4:**
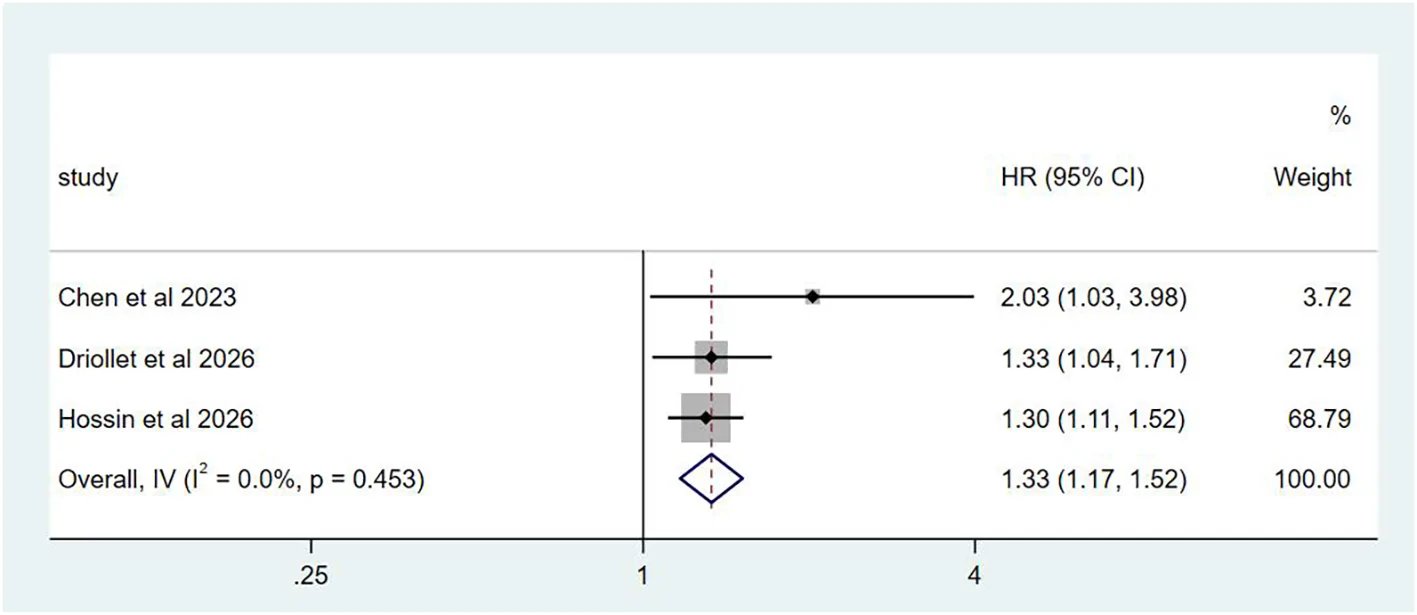
Forest plot of the association between pregestational type 1 diabetes mellitus and the risk of epilepsy in offspring.

Three studies assessed the association between maternal T1DM and epilepsy in offspring. No significant heterogeneity was detected (I² = 0%, *P* = 0.453), and a fixed-effect model was applied. The pooled estimate indicated that pregestational T1DM was significantly associated with an elevated risk of epilepsy in offspring (HR = 1.33, 95% CI [1.17, 1.52], *P* < 0.0001) (**Figure 4**).

### Sensitivity analysis

**Figure 5:**
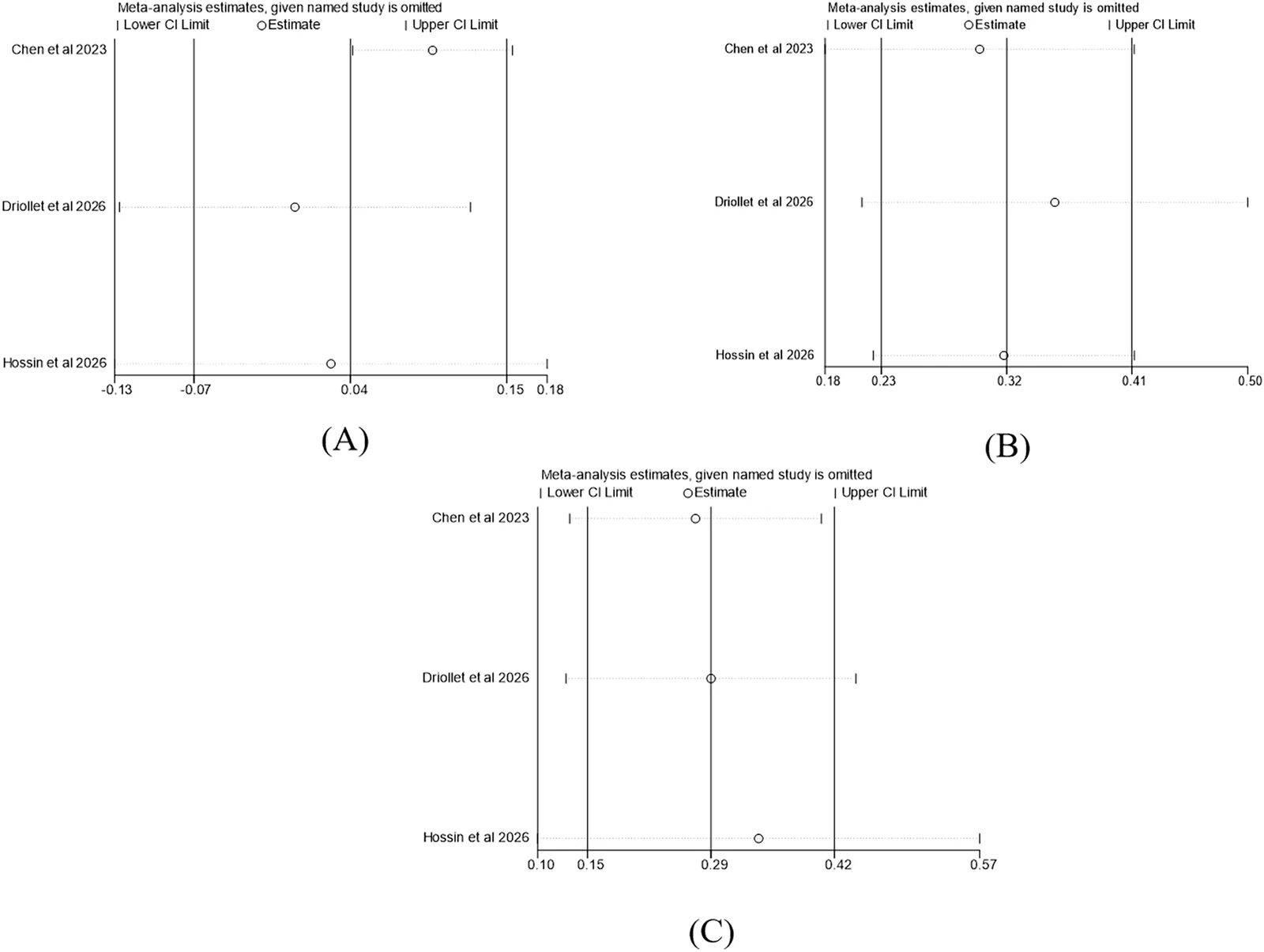
Sensitivity analysis. **(A)** The association between gestational diabetes mellitus and the risk of epilepsy in offspring; **(B)** The association between pregestational type 2 diabetes mellitus and the risk of epilepsy in offspring; **(C)** The association between pregestational type 1 diabetes mellitus and the risk of epilepsy in offspring.

Sensitivity analyses were conducted using the leave-one-out approach, in which the pooled effect size was recalculated after sequentially excluding each individual study. For the GDM–epilepsy association, the results proved unstable: when the study by Chen et al. (18) was excluded, the summary estimate reversed direction and became statistically significant, suggesting that GDM increased the risk of offspring epilepsy (HR = 1.10, 95% CI [1.04, 1.17], *P* = 0.001). In contrast, the sensitivity analyses for pregestational T1DM and T2DM demonstrated robust findings, with no single study unduly influencing the pooled estimates (**Figure 5**).

### Publication bias

**Figure 6:**
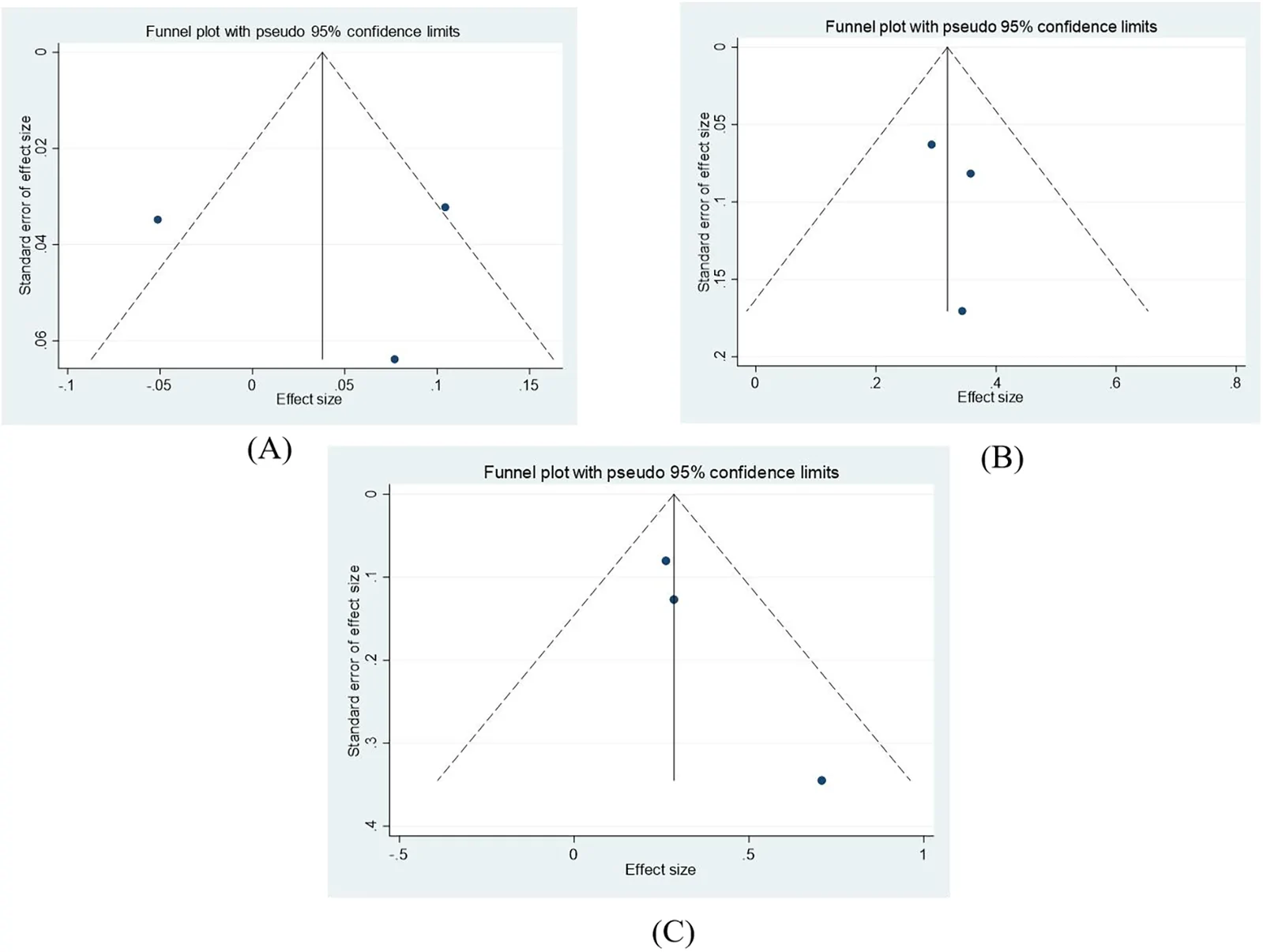
Funnel plot for publication bias. **(A)** The association between gestational diabetes mellitus and the risk of epilepsy in offspring; **(B)** The association between pregestational type 2 diabetes mellitus and the risk of epilepsy in offspring; **(C)** The association between pregestational type 1 diabetes mellitus and the risk of epilepsy in offspring.

Visual inspection of the funnel plot revealed asymmetry in the distribution of effect sizes on both sides of the pooled estimate, suggesting a potential risk of publication bias (**Figure 6**). Although the results of Egger’s test and Begg’s test did not reach statistical significance (**Table 3**), it is critical to emphasize that the number of included studies for each exposure–outcome analysis was fewer than 10, which falls well below the minimum recommended threshold for reliable publication bias testing. Under these conditions, the statistical power of these tests is extremely low, rendering their findings of limited inferential value and insufficient to rule out the presence of publication bias. Consequently, the risk of publication bias cannot be excluded; given that studies with null or negative findings are less likely to be published, the pooled effect estimates—particularly those for significant associations—may be prone to overestimation.

**Table 3:** Publication bias analysis.

Outcome	Egger’s test (*P* value)	Begg’s test (*P* value)
GDM	0.680	0.420
T2DM	0.907	1.000
T1DM	0.342	0.296

T1DM, Type 1 Diabetes Mellitus; T2DM, Type 2 Diabetes Mellitus; GDM, Gestational Diabetes Mellitus.

## Discussion

This systematic review and meta-analysis, synthesizing data from three observational studies comprising a total of 5,287,837 participants, systematically evaluated the associations between distinct subtypes of maternal diabetes—pregestational T1DM, pregestational T2DM, and GDM—and the risk of epilepsy in offspring. The results demonstrated that both pregestational T1DM and T2DM significantly increased the risk of epilepsy in offspring. In contrast, no overall significant association was observed between GDM and offspring epilepsy; however, sensitivity analyses indicated that this null finding was heavily influenced by a single study and lacked robustness.

In this study, the positive associations identified between pregestational T1DM and T2DM and offspring epilepsy risk are consistent with the majority of previous studies (8, 18, 19). The underlying mechanisms may involve a range of pathophysiological alterations within the intrauterine environment. Although maternal hyperglycemia has biological plausibility in affecting fetal neurodevelopment, it is crucial to emphasize that all evidence incorporated in this meta-analysis originates from observational studies; accordingly, these findings reflect statistical associations rather than established causal relationships. Pregestational diabetes is often characterized by chronic hyperglycemia, exposing the fetus to persistently elevated glucose concentrations, which can induce fetal hyperinsulinemia and subsequent hypoglycemia, thereby compromising the energy metabolism and oxygenation status of developing brain tissue (23–25). Moreover, glycemic control in women with preexisting diabetes tends to be highly variable (26, 27). Exposure to hyperglycemia during early pregnancy coincides precisely with the critical window of neural tube closure and early brain structural development, potentially inflicting irreversible structural or functional damage to the central nervous system (28, 29). Maternal hyperglycemia can also elevate oxidative stress levels in fetal brain tissue, promote neuronal apoptosis, and interfere with the expression of synaptic plasticity-related molecules (30). In addition, pregestational diabetes—particularly T1DM—is frequently accompanied by autoimmune disorders, microvascular complications, and placental dysfunction (31–33). These factors may collectively increase the risk of aberrant neurological development in offspring through pathways such as chronic intrauterine hypoxia and excessive release of pro-inflammatory cytokines (25, 34, 35). Notably, the magnitudes of risk elevation for offspring epilepsy were similar between T1DM and T2DM (hazard ratios of 1.33 and 1.38, respectively), suggesting that although the pathophysiological bases of these two diabetes types differ, intrauterine hyperglycemic exposure per se may constitute the core driver of increased risk, rather than diabetes-type-specific factors. Nevertheless, the above mechanistic interpretations should be strictly regarded as exploratory explanations for observational associations, not as definitive conclusions regarding causal pathways; their validation requires further corroboration through basic research and interventional studies.

This study did not identify a statistically significant overall association between GDM and offspring epilepsy. However, this finding warrants cautious interpretation. First, GDM is typically diagnosed in the second or third trimester of pregnancy (commonly between 24 and 28 weeks of gestation) (11), by which time the major structural development of the fetal brain is largely complete, and the duration of hyperglycemic exposure is comparatively short. In contrast, pregestational T1DM and T2DM expose the fetus to hyperglycemia from the moment of conception onward (8). Thus, the timing, intensity, and duration of hyperglycemic exposure during pregnancy are critical determinants of neurodevelopmental outcomes. Second, the degree of hyperglycemia in GDM is generally less severe than that in pregestational diabetes, and most GDM patients achieve satisfactory glycemic control through dietary modification, physical exercise, or insulin therapy, which may attenuate the adverse effects of hyperglycemia on the fetal brain to some extent. Furthermore, while GDM typically resolves after delivery, pregestational diabetes persists postpartum, potentially extending exposure effects into breastfeeding and childhood family environments. This difference in postpartum continuous exposure may also account for the divergent risk profiles observed between the two types of diabetes.

Notably, when the study by Chen et al. (18) was excluded, the pooled effect size for the GDM–epilepsy association reversed direction and became statistically significant. This finding indicates that the current meta-analytic result for GDM may be overly dependent on a single study and lacks robustness. The study by Chen et al. (18) had the largest sample size among the included studies; its original findings might have been substantially influenced by the specific population characteristics, GDM diagnostic criteria, or the particular set of confounders adjusted for. Therefore, it would be premature to conclude definitively that GDM has no effect on offspring epilepsy based solely on the present evidence. Future large-scale prospective studies employing standardized diagnostic criteria are urgently needed to clarify this issue.

From a clinical and public health perspective, although the positive associations identified between pregestational maternal diabetes and offspring epilepsy risk do not imply causation, they nonetheless offer several heuristic insights for clinical practice. First, for women with preexisting diabetes planning pregnancy, optimizing periconceptional glycemic control remains a standard recommendation for safeguarding maternal and fetal health. Although direct evidence that this measure specifically reduces offspring epilepsy risk is currently lacking, given the potential adverse effects of hyperglycemia on fetal multisystem development, rigorous glycemic management is a reasonable and prudent clinical strategy. Second, for offspring of mothers with pregestational diabetes, enhanced long-term neurodevelopmental surveillance—including periodic neurodevelopmental assessments and pediatric neurology referrals—may facilitate early detection of potential problems. However, at present, this should be regarded as a precautionary recommendation based on associative evidence, rather than a proven intervention for epilepsy prevention. Moreover, for GDM, given the instability of the current meta-analytic evidence, it would be inappropriate to infer that intensified diabetes management could mitigate epilepsy risk. Overall, all of the above recommendations should be understood as clinical considerations grounded in limited observational data, not as evidence-based intervention guidelines; their effectiveness awaits confirmation and quantification through rigorously designed interventional studies or individual-patient-data meta-analyses.

In the present meta-analysis, substantial heterogeneity was observed for the GDM outcome analyses, whereas no significant heterogeneity was detected for the T1DM and T2DM analyses. This disparity suggests that the degree of between-study consistency differs across exposure types, and a closer examination of the methodological characteristics of the included studies may help elucidate the sources of heterogeneity. First, with respect to follow-up duration, although all studies covered the childhood-to-adolescence period, during which epilepsy incidence peaks, the exact start and end points of follow-up were not entirely consistent across studies, potentially affecting the cumulative estimation of incidence, particularly for epilepsy subtypes with later age of onset. Longer follow-up tends to capture a more complete spectrum of epilepsy cases, which may partly explain why the GDM effect estimate reported by Hossin et al. (8) was lower than those of the other two studies (18, 19), as extended follow-up might dilute the relative effect of early exposure or introduce additional confounding. However, in the absence of original data stratified by follow-up time, this hypothesis could not be quantitatively verified in the current meta-analysis. Second, regarding outcome definitions, all three studies relied on large national or regional health registries and used International Classification of Diseases (ICD) codes to identify epilepsy cases. Although this standardized coding approach carries some risk of misclassification or under-ascertainment, it provides reasonable comparability across studies and a fundamental basis for pooling. Each study required at least two outpatient diagnoses or one inpatient diagnosis, further enhancing the specificity of outcome definitions. Thus, despite being conducted in different countries, the operational definitions of epilepsy were largely consistent across studies. Third, with respect to population characteristics, the three studies originated from China, Canada, and Sweden, which differ in genetic backgrounds, lifestyles, healthcare accessibility, and baseline epilepsy incidence rates. Temporal changes in epilepsy diagnostic awareness and healthcare resource availability may also introduce period effects. Finally, regarding covariate adjustment, the sets of confounding factors incorporated varied considerably across studies. This diversity in adjustment strategies reflects differences in the richness of health registry data across regions and implies that the degree of control for potential confounders differed, potentially exerting differential effects on the direction of effect estimates. The substantial heterogeneity observed in the GDM analysis likely stems from the combined influence of follow-up duration, population characteristics, and covariate adjustment protocols. In contrast, because T1DM and T2DM are diagnosed prior to pregnancy with more uniform diagnostic criteria, are chronic in nature, and are generally more severe, their effects appear more stable across studies, and no significant heterogeneity was detected. Nevertheless, the uncertainty arising from these methodological differences should be carefully considered when interpreting all pooled results. Given that only three studies were included for each exposure–outcome pair—well below the minimum number required for statistically meaningful subgroup meta-analyses—we were unable to perform formal subgroup comparisons by study design, follow-up duration, or outcome definition. As an alternative, we assessed the robustness of pooled estimates through leave-one-out sensitivity analyses. Although this approach does not directly resolve the sources of heterogeneity, it allows identification of individual studies with disproportionate influence on the overall effect. We found that the GDM pooled result was highly sensitive to the study by Chen et al. (18), suggesting that future updates, once more studies are available, should report results both including and excluding this study.

This study has several additional limitations. First, the number of included studies is limited, and all are observational cohort studies inherently subject to residual confounding. Factors such as maternal body mass index, prepregnancy hypertension, smoking, educational level, and perinatal complications were controlled to varying degrees across original studies, which may have affected the accuracy of effect estimates. Residual confounding represents a major methodological challenge that cannot be fully circumvented. Some studies adjusted for prepregnancy BMI, hypertensive disorders, and socioeconomic status, whereas others additionally adjusted for deeper potential confounders, such as parental history of neuropsychiatric disorders. This inconsistency in adjustment schemes itself reflects differences in the understanding of confounding structures across studies, and no single study could fully exclude all unknown or unmeasured confounders. Of particular concern, adjustment for genetic confounders such as parental neuropsychiatric history—given that epilepsy has a hereditary component and that parents with diabetes and parents with epilepsy may share incompletely observed overlapping characteristics in the general population—may significantly influence effect estimation. Therefore, the positive associations observed between maternal diabetes (especially pregestational subtypes) and offspring epilepsy may be partially attributable to residual confounding rather than solely to direct intrauterine hyperglycemic effects. When interpreting the clinical implications of these associations, it is essential to distinguish carefully between statistical association and causal effect. Second, the definitions of epilepsy outcomes varied across studies, which may have introduced information bias. Third, the original studies predominantly originated from high-income countries (Canada and Sweden) and one upper-middle-income country (China); consequently, the generalizability of the findings to low- and middle-income countries may be limited. Fourth, this meta-analysis was based on published aggregate data rather than individual participant data, precluding more detailed subgroup analyses (e.g., stratified by glycemic control levels, duration of diabetes, or medication use during pregnancy), which limits in-depth exploration of dose–response relationships and potential effect modifiers. Fifth, the included studies did not sufficiently distinguish the continuous postpartum influence of maternal diabetes status on offspring epilepsy, which may confound the effects of intrauterine exposure and postnatal environmental exposure. Sixth, the assessment of publication bias was severely constrained by methodological limitations. Although we employed funnel plots, Egger’s test, and Begg’s test for a comprehensive evaluation, the number of original studies included in this meta-analysis was exceedingly small. With such a limited sample size, visual inspection of funnel plot symmetry is inherently subjective, and the statistical power of Egger’s and Begg’s tests is critically insufficient; consequently, non-significant results from these tests are highly prone to false-negative errors. Therefore, we are unable to draw any meaningful statistical inference regarding publication bias, nor can we rule out the possibility that the absence of unpublished null findings has distorted the pooled effect estimates. This risk should be fully taken into account when interpreting the results of this meta-analysis.

## Conclusion

In summary, this meta-analysis suggests that pregestational T1DM and T2DM may increase the risk of epilepsy in offspring. In contrast, for GDM, the current analytical evidence remains inconclusive, with notable between-study inconsistency; although the pooled effect was not statistically significant, the result proved highly unstable under sensitivity testing. Given the limited number of included studies and the inability to fully exclude residual confounding, the above findings—particularly those pertaining to GDM—should be regarded as exploratory rather than definitive causal inferences, and their interpretation warrants considerable caution. In clinical practice, it is essential to strengthen periconceptional glycemic management for women with pregestational diabetes and to implement long-term neurodevelopmental monitoring for high-risk offspring. Future high-quality, standardized, and large-scale prospective studies are urgently needed to validate these findings and to further elucidate the underlying biological mechanisms and potential intervention strategies.

## Data Availability

The datasets presented in this study can be found in online repositories. The names of the repository/repositories and accession number(s) can be found in the article/**Supplementary Material**.

## Supplementary material

Supplementary Table 1 Search strategy.
