# Supplementary material for: The association between maternal diabetes and the risk of epilepsy in offspring: a systematic review and meta-analysis
Source: Front Endocrinol (Lausanne). 2026 Sep 11;17:1960502. doi: 10.3389/fendo.2026.1960502 (PMC13612357; doi:10.3389/fendo.2026.1960502)
Supplement: Supplementary file 1 [file DataSheet1.docx]

| **Supplementary Materials Table S1. Search strategy** | |
| --- | --- |
| Pubmed | ("Diabetes, Gestational"[Mesh] OR "Diabetes Mellitus"[Mesh] OR "Pregnancy in Diabetics"[Mesh] OR gestational diabetes mellitus[tiab] OR GDM[tiab] OR gestational diabetes[tiab] OR diabetes mellitus[tiab] OR pre-gestational diabetes[tiab] OR preexisting diabetes[tiab] OR type 1 diabetes[tiab] OR T1DM[tiab] OR type 2 diabetes[tiab] OR T2DM[tiab] OR maternal diabetes[tiab] OR diabetic pregnancy[tiab]) AND ("Epilepsy"[Mesh] OR "Seizures"[Mesh] OR epilepsy[tiab] OR epilep*[tiab] OR seizure*[tiab] OR convuls*[tiab]) AND ("Offspring"[Mesh] OR "Child"[Mesh] OR "Infant"[Mesh] OR "Pregnancy"[Mesh] OR offspring[tiab] OR child*[tiab] OR infant*[tiab] OR neonat*[tiab] OR newborn[tiab] OR progeny[tiab] OR fetal[tiab] OR foetal[tiab] OR prenatal[tiab] OR in utero[tiab]) |
| The Cochrane Library | #1 [mh "Diabetes, Gestational"] OR [mh "Diabetes Mellitus"] OR [mh "Pregnancy in Diabetics"] #2 gestational diabetes mellitus:ti,ab,kw OR GDM:ti,ab,kw OR gestational diabetes:ti,ab,kw OR diabetes mellitus:ti,ab,kw OR pre-gestational diabetes:ti,ab,kw OR preexisting diabetes:ti,ab,kw OR type 1 diabetes:ti,ab,kw OR T1DM:ti,ab,kw OR type 2 diabetes:ti,ab,kw OR T2DM:ti,ab,kw OR maternal diabetes:ti,ab,kw OR diabetic pregnancy:ti,ab,kw #3 #1 OR #2 #4 [mh Epilepsy] OR [mh Seizures] #5 epilepsy:ti,ab,kw OR epilep*:ti,ab,kw OR seizure*:ti,ab,kw OR convuls*:ti,ab,kw #6 #4 OR #5 #7 [mh Offspring] OR [mh Child] OR [mh Infant] OR [mh Pregnancy] #8 offspring:ti,ab,kw OR child*:ti,ab,kw OR infant*:ti,ab,kw OR neonat*:ti,ab,kw OR newborn:ti,ab,kw OR progeny:ti,ab,kw OR fetal:ti,ab,kw OR foetal:ti,ab,kw OR prenatal:ti,ab,kw OR "in utero":ti,ab,kw #9 #7 OR #8 #10 #3 AND #6 AND #9 |
| Embase | #1 'gestational diabetes'/exp OR 'diabetes mellitus'/exp OR 'pregnancy diabetes'/exp #2 'gestational diabetes mellitus':ti,ab,kw OR GDM:ti,ab,kw OR 'gestational diabetes':ti,ab,kw OR 'diabetes mellitus':ti,ab,kw OR 'pre-gestational diabetes':ti,ab,kw OR 'preexisting diabetes':ti,ab,kw OR 'type 1 diabetes':ti,ab,kw OR T1DM:ti,ab,kw OR 'type 2 diabetes':ti,ab,kw OR T2DM:ti,ab,kw OR 'maternal diabetes':ti,ab,kw OR 'diabetic pregnancy':ti,ab,kw #3 #1 OR #2 #4 'epilepsy'/exp OR 'seizure'/exp #5 epilepsy:ti,ab,kw OR epilep*:ti,ab,kw OR seizure*:ti,ab,kw OR convuls*:ti,ab,kw #6 #4 OR #5 #7 'offspring'/exp OR 'child'/exp OR 'infant'/exp OR 'pregnancy'/exp #8 offspring:ti,ab,kw OR child*:ti,ab,kw OR infant*:ti,ab,kw OR neonat*:ti,ab,kw OR newborn:ti,ab,kw OR progeny:ti,ab,kw OR fetal:ti,ab,kw OR foetal:ti,ab,kw OR prenatal:ti,ab,kw OR 'in utero':ti,ab,kw #9 #7 OR #8 #10 #3 AND #6 AND #9 |
| Web of Science | TS=("gestational diabetes mellitus" OR "gestational diabetes" OR GDM OR "diabetes mellitus" OR "pre-gestational diabetes" OR "preexisting diabetes" OR "type 1 diabetes" OR T1DM OR "type 2 diabetes" OR T2DM OR "maternal diabetes" OR "diabetic pregnancy") AND TS=(epilepsy OR epilep* OR seizure* OR convuls*) AND TS=(offspring OR child* OR infant* OR neonat* OR newborn OR progeny OR fetal OR foetal OR prenatal OR "in utero") |
